# Chemical Analysis and Cytotoxic and Cytostatic Effects of Twelve Honey Samples Collected from Different Regions in Morocco and Palestine

**DOI:** 10.1155/2019/8768210

**Published:** 2019-05-27

**Authors:** Hamada Imtara, Abdalsalam Kmail, Soumaya Touzani, Mira Khader, Hadeel Hamarshi, Bashar Saad, Badiaa Lyoussi

**Affiliations:** ^1^Physiology-Pharmacology, University of Fez, P.O. Box 1796, Fez Atlas, Fez, Morocco; ^2^Qasemi Research Center, Al-Qasemi Academic College and Faculty of Arts and Sciences, Arab American University, Palestine, P.O. Box 240, Jenin, State of Palestine

## Abstract

The aim of this* in vitro* study is to characterize the phenolic compounds of twelve honey samples collected from different locations in Palestine (H1-6) and Morocco (H7-12) and to evaluate their cytotoxic and cytostatic effects in cells from the human colorectal carcinoma cell line HCT-116 and breast cancer cell line MCF-7. Quantitative HPLC analysis revealed nine phenolic compounds in three Moroccan honey samples, namely, syringic acid, tannic acid, caffeic acid, ferulic acid, coumaric acid, gallic acid, rosmarinic acid, epicatechin, and pyrogallol. Syringic acid, abundant in numerous types of honey with strong antioxidant capacities, was present at values ranging between 0.10 mg/100 g and 1.24 mg/100 g of Daghmos (H11) and Kabbar (H10) samples, respectively. No significant reductions in cell viability were observed in both cell lines treated with the Palestinian samples as measured with MTT assay. Significant cytostatic effects were after treatment of HCT cells with Morar honey H1 with IC50 of 1789 *μ*g/ml. Three Moroccan samples, H7 (Zaâtar), H9 (Bochnikha), and H10 (Kabbar), showed slight, but significant cytostatic effects in HCT cells. A strong correlation was observed between cytostatic activity of MCF cells and antioxidant content (phenols, flavonoids, and flavonol). Furthermore, a strong negative correlation was detected between the cytostatic activity in HCT cells and the contents of syringic acid (r= -0.756) and tannic acid (r= -0.610). These results indicate that the traditionally known anticancer effects of honey might be mediated in part through cytostatic effects.

## 1. Introduction

Cancer diseases are considered as one of the most important health problems and they are the second cause of death worldwide [1–3]. According to the World Health Organization, more than 14 million people developed cancer in 2012 and the number of people dying of cancer was about 8.2 million [3]. The breast cancer and colorectal cancer are the second and third leading causes of cancer related deaths worldwide, respectively [4]. Breast cancer is the most common type of cancer among Palestinian and Moroccan, females. Colorectal cancer is the third type of cancer affecting the population in both countries [5, 6].

The use of chemotherapy drugs is associated with unwanted side effects and these drugs may lose effectiveness over time due to the resistance of their cancerous cells [7]. In order to improve the effectiveness of treatment of cancer patients and to reduce the side effects of anticancer drugs, researchers focused on the search for alternative natural or adjuvant that confer maximum effects and are less harmful for cancer patients [8]. Alternative natural products, including bee products, are an important source of therapeutic agents because they contain relatively high amounts of phenolic compounds that play a role in the suppressing of carcinogenesis [8, 9]. One of bee products that have become an important component of alternative natural products is honey, a food bank for health and important for the treatment of many diseases [10, 11]. Numerous of biological and pharmacological properties of honey have been noted including antibacterial, antifungal, anti-inflammatory, antioxidant, immunomodulatory, antiviral, wound healing, hepatoprotective, antidiabetic, hypoglycemic, and antihypertensive effects [12, 13]. These activities are the results of honey-derived compounds, such as phenols, flavonoids, vitamins, trace elements, proteins, amino acids, and sugars content [9].

Honey was documented to exhibit inhibitory activity of carcinogenesis (initiation, proliferation, and progression) [8]. Many studies and researches have shown the mechanisms practiced by some types of honey on HCT and MCF cancer cells. Among these mechanisms, antagonizing estrogen activity, inhibiting cell proliferation, inducing apoptosis, reducing mitochondrial membrane potential arrests cell cycle, reducing mitochondrial membrane potential, increasing generation of reactive oxygen species (ROS), depleting intracellular nonprotein thiols, inducing DNA damage, and suppresing inflammation, these mechanisms vary from honey to another [7, 14–16].

The cellular and molecular action mechanisms of the traditionally claimed anticancer properties of the honey from Palestine and Morocco are so far not well elucidated. Here, we aim to assess the cytotoxic and cytostatic properties of samples collected from different botanical origin in Palestine and Morocco and to identify selected phenolic compounds that may be the cause of this activity. A strong correlation was seen between cytostatic activity of MCF cells and antioxidant content and a strong negative correlation between cytostatic activity and syringic acid (r = -0.756) and tannic acid (r = -0.610) was seen with HCT cells. These results indicate that the traditionally claimed anticancer effects of honey might be mediated in part through cytostatic effects.

## 2. Material and Methods

### 2.1. Honey Samples

Six local Palestinian (H11-H6) and six local Moroccan (H7-H12) honey samples were purchased from beekeeper in each country, stored at room temperature (22–24°C) in airtight plastic containers until analysis, and labelled based on the commercial containers descriptions. Also, the data of the previous work of the same sample [13] was used in order to work correlations and multivariate analysis (Table 1).

### 2.2. Extraction and Identification of Phenolic Compounds of Honey by HPLC

Honey samples were subjected to base hydrolysis and extracted with ethyl acetate (liquid-liquid extraction) as described in [17]. In brief, 10 g of each honey sample were dissolved in acidified distilled water (pH=3.5), and phenolic compounds were extracted with ethyl acetate. The phenolic extracts were passed in rotavapor in order to remove the solvent. The obtained dry phenolic extracts were then dissolved in 5 ml of methanol (HPLC grade). The HPLC analysis was carried out at 280 nm for phenolic compounds and operated at 30°C using a C18 column (4,6 × 150 mm) × 5 mm in a Thermo Fisher apparatus, and the injected volume was 20 *μ*L. Solvent (A) was MeoH/Acet (50%- 50%) and solvent (B) was acidified water H_3_PO_4_^−^ (0.2%), at a flow rate of 1 mL/min. The elution gradient was 30–95% (B) for a total run time of 53 min, starting from 5% solvent A and 95% solvent B, decreasing to 65% solvent B over 30 min, and to 30% over 10 min. The following pure compounds were used as external standards: syringic acid, tannic acid, caffeic acid, ferulic acid, coumaric acid, gallic acid, rosmarinic acid, epicatechin, and pyrogallol; all standards were dissolved in methanol and injected under the same chromatographic conditions as the honey extracts. Phenolic compounds of honey extracts were identified by comparing their retention times with those of pure standards. The results obtained in mg/100g of honey.

### 2.3. Cell Culture

Human colorectal carcinoma cell line HCT-116 (ATCC® CCL-247™) and breast cancer cell line MCF-7 (ATCC® HTB-22™) were grown in DMEM-5671 with a high glucose content (4.5 g/l), supplemented with 10% vol/vol inactivated fetal calf serum (FCS), 1% nonessential amino acids, 1% glutamine, 100 U/ml penicillin, and 10 *μ*g/ml streptomycin. The pH of the media for growing cells was maintained at 7.4 under 5% CO2 at 37°C.

*Cytotoxic and Cytostatic Effects in Monoculture System*. For the cytotoxic and cytostatic assays, 20,000 cells/100 *μ*l and 5,000 cells/100 *μ*l medium were seeded in 96-microtiter plates, respectively. Twenty-four hours later, cells were incubated with increasing concentrations of honey solutions (0-2000 *μ*g/ml of culture media) for 24 hours and 72 hours for cytotoxic and cytostatic assays, respectively. Then cell viability was measured using the MTT assay. Cell Viability was defined as the ratio (expressed as a percentage) of absorbance of treated cells to untreated cells (control).

### 2.4. MTT Assay

MTT (3-(4,5-Dimethylthiazol-2-yl)-2,5-diphenyltetrazolium bromide) cell viability assay was carried out as described by Kaadan et* al*. [18]. Briefly, twenty-four hours after cell seeding, cells were exposed to varying concentrations of the honey samples (0-2000 *μ*g/ml of culture media) for 24 hours in cytotoxic test and for 72 hours in cytostatic test at 37°C. Following the removal of the media from each well, cells were washed in phosphate buffered saline (PBS) and incubated in serum-free medium to which MTT (0.5 mg/ml) was added to each well (100 *μ*l),and incubated for four hours in the dark. Then the cells were washed with PBS and incubated for 15 minutes with 100 *μ*L of acidic isopropanol (0.08 N HCl) to dissolve the formazan crystals occluded in the mitochondria. The absorbance of the MTT formazan was determined at 570 nm in an ELISA reader. Cell viability was defined as the ratio (expressed as a percentage) of absorbance of treated cells to untreated control cells (Control).

### 2.5. Statistical Analysis

The statistical analysis was performed by ANOVA through the GraphPad Prism 6 program and using the Tukey post hoc test at P<0.05. Correlations between bioactive compounds and cytostatic activity were achieved by Pearson correlation coefficient (r) at a significance level of 95% (P<0.01). The PCA analyses of data were accomplished using MultBiplot64.

## 3. Results and Discussion

### 3.1. Identification of Phenolic Compounds of Honey Samples by HPLC

Polyphenols belong to a major group of secondary metabolites synthesized by the plant kingdom. They are naturally present in our food in various forms such as flavonoids, phenolic acids, stilbenes, and lignans [19, 20]. Numerous scientific papers have documented the beneficial health properties of polyphenols. These include, but not limited to, antiseptic, anticancer, anti-inflammatory, and antioxidant [21]. In order to extract the phenolic compounds in tested honey samples, we applied liquid-liquid extraction method with ethyl acetate as solvent [22].

The polyphenolic composition of honeys varies depending on the source that is pollinated and to the geographical and climatic conditions [23, 24]. A study conducted on the total phenolic content in samples of Palestinian honey collected from different regions and flora in Palestine showed that the phenolic content ranges between 26.96 and 70.73 mg equivalence per 100g of honey [10]. Results obtained here of the quantitative analysis of phenolic compounds in honey samples are presented in Table 2. Nine phenolic compounds, namely, syringic acid, tannic acid, caffeic acid, ferulic acid, coumaric acid, gallic acid, rosmarinic acid, epicatechin, and pyrogallol were identified in three Moroccan honey types; Zaâtar (H7), Kabbar (H10), and Arbousie (H12). The syringic acid abundant in numerous types of honey is known by antioxidant capacities to eliminate free radicals [25], in the present work the values of syringic acid ranging between 0.10 mg /100 g of honey in sample H11 and 1.24 mg/100 g of honey in Kabbar sample H10 (Table 2). No measurable concentrations were detected in four samples (Morar akhdar (H2), Multifloral (H5), Sader (H6), and Limon (H8)).

Phenolic acids are widely used as chemical indicators of the botanical origin of honey samples [26, 27]. For example, the caffeic acid, p-coumaric acid, and ferulic acid—in variable proportions—are typical for chestnut, lavender, sunflower, and acacia honeys [28]. Also, it is interesting to note that major phenolic compounds in honeys are formed from monophenols such as ferulic acid, which was not detected in 3 of the samples analyzed (Morar akhdar (H2), Limon (H8), and Daghmos (H11)), and it was present in Multifloral (H5), Arbousie (H12), and Kabbar (H10) honeys with amounts of 1.93 mg/100 g; 1.83 mg /100 g; 1.70 mg /100g, respectively, while the most important contents of coumaric acid were recorded in Daghmos (H11), Bouchnikha (H9), and Multifloral (H5) honeys with values of 8.20 mg/100 g, 4.42 mg /100 g, and 2.75 mg/100 g, respectively. Concerning the caffeic acid (CA), which is a representative phenolic compound found in many natural resources such as honey [21], CA has many biological activities, including antioxidant, anticancer, and antidiabetic effects [29, 30]. Highest CA value was measured in Daghmos honey (H11), while the other samples had very low values compared to the H11. Other phenolic acids have been proposed; of the botanical origin was the rosmarinic acid indicated for Zaâtar honey [26]. In the present work rosmarinic acid content in Zaâtar honey (H7) was 0.02 mg/100 g. It is very low compared to the honey of Bouchnikha (H9) with a value of 1.38 mg /100 g of honey. The presence of rosmarinic acid in Zaâtar honey is in agreement with the study conducted by Paula et al. on the honey of thyme (Zaâtar) [26].

Tannic acid is a phenolic acid which is found in several foods and beverages playing a role in the organoleptic and nutritional qualities of products [31]. It has been shown to possess many properties including antimutagenic, antioxidant, and anticarcinogenic [32]. In the present work, five samples were devoid of this compound (Morar akhdar (H2), Zohif (H3), Sader (H6), Limon (H8), and Daghmos (H11)). In addition, the Multifloral honey (H5) is the richest in tannic acid with a content of 5.62 mg/100 g of honey followed by Arbousie (H12) and Kabbar (H10) honeys with contents of 5.54 mg/100 g and 5.39 mg/100 g honey, respectively.

Ahmed et* al*. have identified the gallic acid with different quantities in two Malaysian honeys [17]. The values obtained for gallic acid show significant differences varying between 0.35 mg/100 g and 18.42 mg/100 g of honey. The gallic acid consisting of a trihydroxylated phenolic structure is also known for its antioxidant, anti-inflammatory, antimutagenic, anticancer, and cardioprotective potential [21, 33] and has been identified in many honey samples [34]. Other phenolic compounds present in honey are the pyrogallol which had antitumor activity on human cell lines [21] and was most abundant in honey H12 (Arbousie) with a value of 16.34 mg/100 g. This value was also the highest content compared to other compounds identified in the same sample. The minimum value of pyrogallol was presented by the honey H6 (Sader) with a value 0.31 mg/100 g.

Finally, epicatechin is known by its anticancer activities [35]. The Sader honey (H6) had the lowest value (0.18 mg/100g) and H7 (Zaâtar) had the highest value (6.42 mg/100g), while the Morar (H1), Morar akhdar (H2), Zohif (H3), Rabat (H4), Limon (H8), and Bochnikha (H9) samples did not show detectable levels in this compound. The results of epicatechin in this study are lower than those reported by Islam et al. and higher than the results reported by Pernaet* al*. [36, 37]. The recent studies of phenolic compounds in honey reported that syringic acid, caffeic acid, ferulic acid, coumaric acid, gallic acid, rosmarinic acid, epicatechin, and pyrogallol are present in many honey samples with different proportion [38, 39]. The values of these phenolic compounds found in the present samples were within the ranges reported for honeys by other studies [36–41]. The observed variations in type and quantity of phenolic compounds in honey samples were possibly because of the different floral sources of honeys as well as influences of climatic and nature of soil [42]. Concerning the tannic acid and according to the author's knowledge, this is the first time this compound has been identified in honey. The results of tannic acid in the present work are not consistent with the study conducted by Afroz et* al*. [43]. Such difference may be due to the fact that he used only one sample in his work.

### 3.2. Cytotoxic and Cytostatic Effects of Honey in Monocultures Cell from the Human HCT-116 and MCF-7 Cell Lines

The research for new natural anticancer drugs is one of the main objectives of scientific research. As part of this work, the strategy adopted was the search for compounds in honey that exerts antiproliferative effects (cytostatic effects) in cancer cells at noncytotoxic concentrations. Whatever the mechanism of action of honey on cancer cells, the results obtained show that anticancer activities detected by MTT assays on cancer cells line HCT and MCF are not similar.

**Figure 1:**
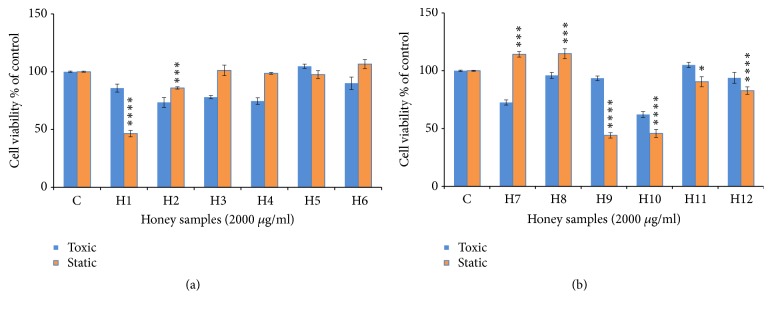
Cytostatic and cytotoxic activity of honey samples (concentration = 2000 *μ*g/ml) in HCT-116 cells. (a) Honey samples from Palestine; (b) honey samples from Morocco. The values are presented in mean ± SD.* ∗ p* <0.05;* ∗∗ p* <0.01;* ∗∗∗ p* <0.001;* ∗∗∗∗p* <0.0001.

**Figure 2:**
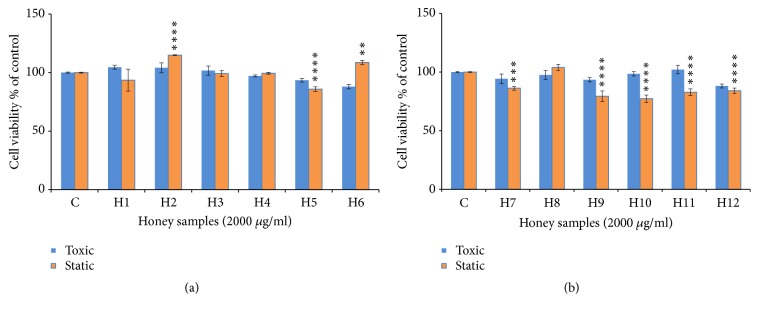
Cytostatic and cytotoxic activity of honey samples (concentration = 2000 *μ*g/ml) in MCF-7 cells. (a) Honey samples from Palestine; (b) honey samples from Morocco. The values are presented in mean ± SD.* ∗ p* <0.05;* ∗∗ p* <0.01;* ∗∗∗ p* <0.001;* ∗∗∗∗p* <0.0001.

**Figure 3:**
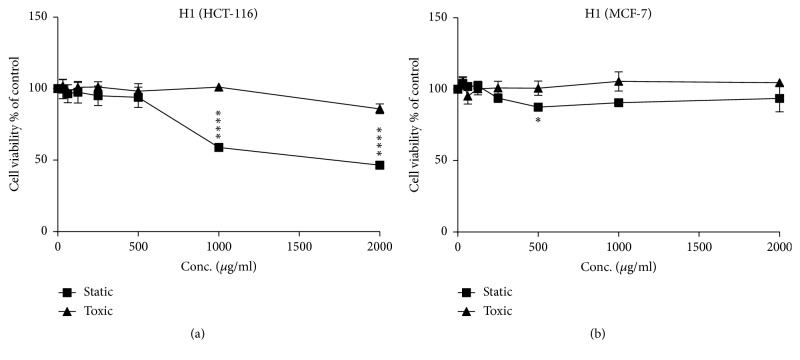
Cytostatic and cytotoxic activity of sample H1 (concentration ranging from 0 to 2000 *μ*g/ml) in HCT and MCF-7 cells. (a) HCT-116 cells line; (b) MCF-7 cells line. The values are presented in mean ± SD.* ∗ p* <0.05;* ∗∗ p* <0.01;* ∗∗∗ p* <0.001;* ∗∗∗∗p* <0.0001.

**Figure 4:**
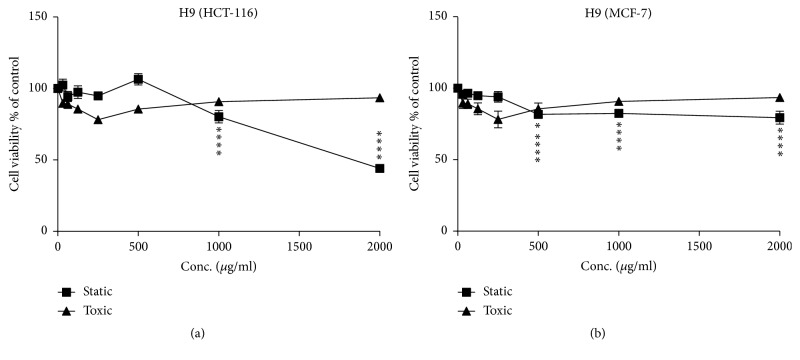
Cytostatic and cytotoxic activity of sample H9 (concentration ranging from 0 to 2000 *μ*g/ml) in HCT and MCF-7 cells. (a) HCT-116 cells line; (b) MCF-7 cells line. The values are presented in mean ± SD.* ∗ p* <0.05;* ∗∗ p* <0.01;* ∗∗∗ p* <0.001;* ∗∗∗∗p* <0.0001.

**Figure 5:**
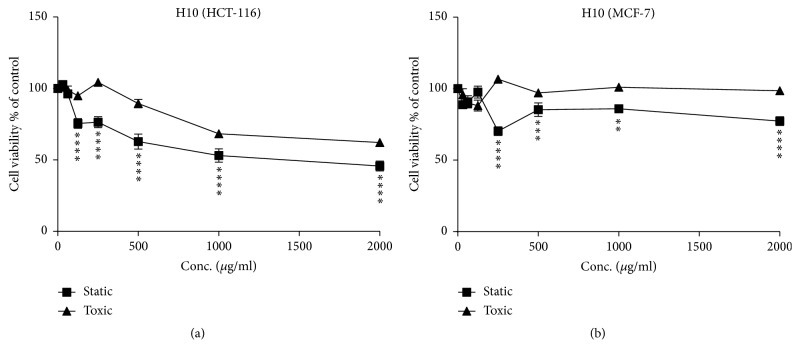
Cytostatic and cytotoxic activity of sample H10 (concentration ranging from 0 to 2000 *μ*g/ml) in HCT and MCF-7 cells. (a) HCT-116 cells line; (b) MCF-7 cells line. The values are presented in mean ± SD.* ∗ p* <0.05;* ∗∗ p* <0.01;* ∗∗∗ p* <0.001;* ∗∗∗∗p* <0.0001.

Figures 1 and 2 show the cytostatic and cytotoxic results of the samples tested on HCT-116 cells. The Morar (H1), Morar akhdar (H2), Bochnikha (H9), Kabbar (H10), Daghmos (H11), and Arbousie (H12) honeys showed an antiproliferative activity on HCT cells at sample concentration of 2000 *μ*g/ml. The honey sample of Kabbar (H10) showed the highest cytotoxic effects with 42% reduction in cell viability compared to untreated control cells. The most significant samples are those that exhibit cytostatic effects at noncytotoxic concentrations. Significant cytostatic effects were seen after treatment of HCT cells with Morar honey (H1) sample from Palestine (Figure 3(a)) with IC50 of 1789 *μ*g/ml (Table 3). Also significant cytostatic effects at none-cytotoxic concentrations were observed after treatment of HCT cells with Bochnikha (H9) and Kabbar (H10) honeys from Morocco (Figures 4(a) and 5(a)) with IC50 of 1863 and 1651 *μ*g/ml, respectively (Table 3). These samples having cytostatic effects were found to be rich in phenolic compounds [13]. Our results are in agreement with many published studies that reported cytostatic effects of honey in colon cancer cells and also suggested that these effects were dependent on the level of phenolic content [8, 16, 44]. In contrary, the honey samples of Zaâtar (H7) and Limon (H8) increased HCT cells proliferation (Figure 1(b)). These results can be explained by the level of hydrogen peroxide of these samples. Hydrogen peroxide was reported to be responsible for the proliferative effect of honey in cancer cells [45]. The results in the present study were similar to other studies [15, 16].

Many authors have shown that the treatment of MCF cells with honey caused an inhibition of cell growth by following mechanisms: antagonizing estrogen activity, inducing apoptosis, and reducing mitochondrial membrane potential [8, 46, 47]. The samples of Multifloral (H5), Zaâtar (H7), Bochnikha (H9), Kabbar(H10), Daghmos (H11), and Arbousie (H12) showed cytostatic activity on MCF cells lines (Figure 2). In contrary, the honey samples of Morar akhdar (H2) and Sader (H6) had a proliferation effect in MCF cells (Figure 2(a)). However, the samples of Morar (H1), Zohif (H3), Rabat (H4), and Limon (H8) did not show any cytostatic effect on MCF cells lines up to a maximum concentration of 2000 *μ*g/ml tested. The results in the present study were similar to other studies [14, 48].

### 3.3. Correlations and Multivariate Analysis

Many studies report a strong correlation between honey color, phenols content, and flavonoids content [10, 13]. In general, darker honeys contain higher amounts of phenols than lighter honeys (Table 1). This theory is consistent with our results, which were explained in another work on the same samples [13]. The results of these correlations were also added in Table 4.

Table 4 depicts the correlations in the samples analyzed in the present work. A strong positive correlation between color and pyrogallol (r = 0.629) and with coumaric acid (r = 0.638) was observed. Amiotet* al*. showed that the yellow color appears to be related to the concentration of flavonoids whereas the intensity of the amber color depends on the phenolic acid content [49]. Still through the results in Table 4, a strong correlation between cytostatic activity of MCF cells and antioxidant content (phenols, flavonoids, and flavonol) is represented by the negative value (r = -0.802; r= -0.817; r= -0.725, respectively). Numerous authors have showed that the anticancer activity of honey can be attributed to the presence of flavonoids and phenols compounds [50, 51]. Since the phenols content governs honey color, a strong negative correlation was observed in the case of color with cytostatic activity of the same cells line (r = -0.815). As a result, the color of honey can be a visual indicator for the selection of honeys with high cytostatic activity on MCF cells. The correlation between bioactive molecules and cytostatic activity can also be seen in the same case of MCF cells, where a good correlation has been demonstrated with the syringic acid, tannic acid, ferulic acid, coumaric acid, and pyrogallol, with values r=-0.619,r=-0.703, r=-0.629, r= -0.591, and r=-0.589, respectively. Phenolic compounds are phytochemicals which are similar to mammalian estrogen and can bind to their receptors. They have an estrogenic or antiestrogenic effect and depend on certain factors such as the concentration [46, 52]. The cytostatic activity of honey on MCF cells can be attributed to the presence of these phenolic acids.

In the case of the cytostatic activity on HCT cells, a strong negative correlation between cytostatic activity and syringic acid (r = -0.756) and also the same correlation cytostatic activity and tannic acid (r= -0.610) were observed (Table 4). A study on colon cancer cells confirmed the antiproliferative effect of honey and also revealed that this effect depended on the content of phenolic compounds [44]. This is consistent with our results in the case of syringic acid and tannic acid. The difference in correlation between cytostatic activity of MCF cells and HCT cells with the phenolic compounds may be due to the variability of mechanisms by which each phenolic compound acts on cells. On the other hand, the nature of the cancer cell may play an important role [8].

**Figure 6:**
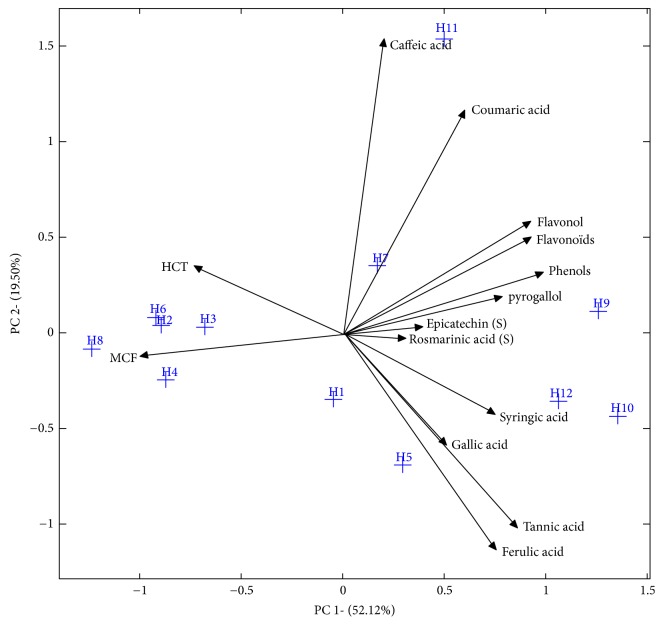
Principal component analysis (PCA) of bioactive compounds and the cytostatic activities.

Regarding the multivariate analyzes, Figure 6 illustrates the integration of the information of the phenolic composition with the cytostatic activity (concentration = 2000*μ*g/ml) of the samples studied using PCA as a powerful tool for reducing scales and concentration of the information in a limited number of so-called principal components. The first two principal components (PC) accounted for 54.37% and 16.49%, respectively, of the information contained in the original data matrix. The first PC, given the component that keeps more information, correlated positively with the total phenols and flavonoids, as well as the rest of the identified phenolic compounds. Therefore, a negative correlation can be observed between the same PC and cytostatic activities.

Considering the similarities of the samples, the first PC allowed the distinction of two groups, each of which had similar characteristics in terms of phenolic composition and cytostatic activities. A first group, composed of samples of Moroccan origin (Bochnikha (H9), Kabbar (H10), and Arbousie (H12)), had high contents of phenolic compounds and thus high cytostatic activities (low % of viability of the cells) in comparison with the other samples. This group is located in the positive part of the graph. The second group consisting of a sample of Moroccan origin (H8: Limon) and Palestinian honey samples (Morar akhdar (H2), Zohif (H3), Rabat (H4), and Sader (H6)) had the opposite characteristics (low phenolic compound content and low cytostatic activity). These samples present in the left part of the graph.

The second PC correlates mainly with caffeic acid and coumaric acid in the positive part, so a negative correlation can be observed in the case of gallic acid and tannic and ferulic acids. Regardless of geographic origin, this second component was able to discriminate honey samples with high content in terms of caffeic acid and coumaric acid from those with high levels of gallic acid, tannic acid, and ferulic acid. This suggests that the negative correlation between the two groups of phenolic compound may be due to the different metabolic pathways in plants which are fed by bees.

## 4. Conclusions

To our knowledge, this is the first extensive investigation of the cytotoxic and cytostatic activities and phenolic compounds by HPLC in honey samples from Palestine and Morocco. Results obtained identify tannic acid for the first time in honey and show a strong correlation between cytostatic activity and antioxidant content in honey samples.

These results indicate that the anticancer effects of honey depend on the plant origin, type, and quantity of the phenolic components contained.

## Figures and Tables

**Table 1:** Local traditional names of six Palestinian (H11-H6) and six local Moroccan (H7-H12) honey samples, their botanical origin, total phenol contents, and color (REF).

Samples	Local traditional name	Botanical origin	Phenols	Honey color
			(mg GAE/100 g)	

H1	Morar	*Centaurea dumulosa*	32.49± 0.08 ^de^	light extra amber
H2	Morar	*Echinops*	33.20 ± 0.29 ^de^	light extra amber
	akhdar	*spinosissimus*		
H3	Zohif	*Thymus capitatus*	42.13 ± 2.17 ^d^	light amber
H4	Rabat	*Conyza bonariensis*	17.97 ± 0.98 ^f^	extra white
H5	Multiforal	*Thymus capitatus*	42.66 ± 2.24 ^d^	light amber
		*Origanum syriacum*		
H6	Sader	*Ziziphus spina-christi*	37.50 ± 2.07 ^d^	light extra amber
H7	Zaâtar	*Thymus vulgaris L*	74.05 ± 1.21 ^b^	Darkamber
H8	Limon	*Citrus limon L*	12.91 ± 0.85 ^f^	water white
H9	Bochnikha	*Ammi visnaga L*	89.53 ± 4.05 ^a^	Amber
H10	Kabbar	*Capparis spinosa*	86.66 ± 1.31 ^a^	Darkamber
H11	Daghmos	*Euphorbia L*	64.54 ± 2.13 ^c^	Darkamber
H12	Arbousie	*Arbutus unedo*	78.45 ± 1.24 ^b^	Amber

**Table 2:** Mean +/- SD of the concentration of phenolic compounds (mg/100g) in honey samples analyzed.

Samples	Syringic acid	Tannic acid	Caffeic acid	Ferulic acid	Coumaric acid	Gallic acid	Rosmarinic acid	Epicatechin	Pyrogallol

H1	0.25 ± 0.09 ^b^	3.36 ± 0.06 ^d^	0.05 ± 0.02 ^b^	0.98 ± 0.16 ^c^	1.73 ± 0.09 ^c^	2.03 ± 1.02 ^cd^	0.25 0.03 ^c^	ND	4.09 ± 0.08 ^d^
H2	ND	ND	0.03 ± 0.00 ^b^	ND	ND	ND ^g^	0.12 ± 0.01 ^d^	ND	0.48 ± 0.06 ^e^
H3	0.12 ± 0.05 ^b^	ND	0.04 ± 0.01 ^b^	0.25 ± 0.04 ^d^	ND	ND ^g^	0.92 ± 0.04 ^b^	ND	ND
H4	0.35 ± 0.08 ^b^	0.60 ± 0.02 ^g^	0.01± 0.00 ^b^	0.45 ± 0.08 ^d^	ND	ND ^g^	ND	ND	ND
H5	ND	5.62 ± 0.15 ^a^	ND	1.93 ± 0.05 ^a^	2.75 ± 0.15 ^c^	18.42 ± 1.27 ^a^	0.15 ± 0.01 ^c^	4.60 ± 0.04 ^b^	ND
H6	ND	ND	0.004 ± 0.00 ^b^	0.02 ± 0.00 ^def^	0.58 ± 0.04 ^c^	0.35 ± 0.02 ^cdf^	0.001 ± 0.00 ^f^	0.18 ± 0.01 ^c^	0.31 ± 0.06 ^e^
H7	0.16 ± 0.02 ^b^	1.20 ± 0.04 ^f^	0.03 ± 0.01 ^b^	0.16 ± 0.04 ^de^	0.72 ± 0.06 ^c^	1.16 ± 0.06 ^cdf^	0.02 ± 0.00 ^e^	6.42 ± 0.16 ^a^	3.65 ± 0.23 ^d^
H8	ND	ND	ND	ND	ND	ND ^g^	ND	ND	ND
H9	0.68 ± 0.01 ^b^	4.04 ± 0.08 ^c^	0.22 ± 0.06 ^b^	0.89 ± 0.08 ^c^	4.42 ± 1.05 ^b^	4.22 ± 0.04 ^c^	1.38 ± 0.09 ^a^	ND	9.65 ± 0.29 ^b^
H10	1.24 ± 0.65 ^a^	5.39 ± 0.09 ^ab^	0.02 ± 0.00 ^b^	1.70 ± 0.04 ^ab^	1.40 ± 0.02 ^c^	3.20 ± 0.23 ^c^	0.06 ± 0.01 ^e^	0.39 0.02 ^c^	3.33 ± 0.84 ^d^
H11	0.10 ± 0.01 ^b^	ND	5.88 ± 1.15 ^a^	ND	8.20 ± 0.54 ^a^	3.78 ± 0.06 ^c^	ND	1.76 ± 0.1 ^c^	6.23 ± 0.07 ^c^
H12	0.32 ± 0.02 ^b^	5.54 ± 0.06 ^a^	0.03 ± 0.01 ^b^	1.83 ± 0.01 ^a^	1.51 ± 0.08 ^c^	7.69 ± 0.84 ^b^	0.07 ± 0.01 ^e^	3.92 ± 1.48 ^b^	16.34 ± 1.13 ^a^

Min-Max	0.10 – 1.24	0.60 – 5.62	0.01 – 5.88	0.02 – 1.93	0.58 – 8.20	0.35 - 18.42	0.001- 1.38	0.18 -6.42	0.31 – 16.34

Average	0.27	4.14	0.53	0.68	1.78	3.40	0.25	1.44	3.67

**Table 3:** IC50 values (*μ*g/mL) of the honey samples were measured in two cell types after 72h at cell density of 5000 cell/well using the MTT test.

Sample/cells	HCT	MCF

H1	1789.3	Over 2000
H9	1862.5	Over 2000
H10	1651	Over 2000

**Table 4:** Pearson correlation coefficients between physicochemical parameters, antioxidant content, and the cytostatic activity.

	Phenols	Flavonoids	Flavonol	Syringic acid	Tannic acid	ferulic acid	Coumaric acid	Pyrogallol	Color

MCF	-0,802^∗∗^	-0,817^∗∗^	-0,725^∗∗^	-0.619^∗^	-0,703^∗^	-0,629^∗^	-0,591^∗^	-0,589^∗^	-0,815^∗∗^
HCT	-	-	-	-0.756^∗∗^	-0,610^∗^	-	-	-	-
Color	0.938^∗∗^	0.950^∗∗^	0.944^∗∗^	-	-	-	0.638^∗^	0.629^∗^	1

∗The correlation is significant at the 0.05 level. ∗∗The correlation is significant at the 0.01 level. -The correlation is not significant.

## Data Availability

The data used to support the findings of this study are available from the corresponding author upon request.

## References

[1] Ma X., Yu H. (2006). Global burden of cancer. *Yale Journal of Biology and Medicine*.

[2] Torre L. A., Bray F., Siegel R. L., Ferlay J., Lortet-Tieulent J., Jemal A. (2015). Global cancer statistics, 2012. *CA: A Cancer Journal for Clinicians*.

[3] WHO (2013). Latest world cancer statistics Global cancer burden rises to 14 . 1 million new cases in 2012: Marked increase in breast cancers must be addressed. *International Agency for Research on Cancer*.

[4] Ferlay J., Soerjomataram I., Dikshit R. (2015). Cancer incidence and mortality worldwide: sources, methods and major patterns in GLOBOCAN 2012. *International Journal of Cancer*.

[5] Elidrissi Errahhali M., Elidrissi Errahhali M., Ouarzane M., Boulouiz R., Bellaoui M. (2017). Cancer incidence in eastern Morocco: cancer patterns and incidence trends, 2005-2012. *BMC Cancer*.

[6] Abu-Rmeileh N. M. E., Gianicolo E. A. L., Bruni A. (2016). Cancer mortality in the west bank, occupied palestinian territory chronic disease epidemiology. *BMC Public Health*.

[7] Fauzi A. N., Norazmi M. N., Yaacob N. S. (2011). Tualang honey induces apoptosis and disrupts the mitochondrial membrane potential of human breast and cervical cancer cell lines. *Food and Chemical Toxicology*.

[8] Erejuwa O. O., Sulaiman S. A., Ab Wahab M. S. (2014). Effects of honey and its mechanisms of action on the development and progression of cancer. *Molecules*.

[9] Viuda-Martos M., Ruiz-Navajas Y., Fernández-López J., Pérez-Álvarez J. A. (2008). Functional properties of honey, propolis, and royal jelly. *Journal of Food Science*.

[10] Imtara H., Elamine Y., Lyoussi B. (2018). Physicochemical characterization and antioxidant activity of Palestinian honey samples. *Food Science & Nutrition*.

[11] Imtara H., Al-Waili N., Bakour M., Al-Waili W., Lyoussi B. (2018). Evaluation of antioxidant, diuretic, and wound healing effect of Tulkarm honey and its effect on kidney function in rats. *Veterinary World*.

[12] Eteraf-Oskouei T., Najafi M. (2013). Traditional and modern uses of natural honey in human diseases: a review. *Iranian Journal of Basic Medical Sciences*.

[13] Imtara H., Elamine Y., Lyoussi B. (2018). Honey antibacterial effect boosting using origanum vulgare L. essential oil. *Evidence-Based Complementary and Alternative Medicine*.

[14] Yaacob N. S., Nengsih A., Norazmi M. N. (2013). Tualang honey promotes apoptotic cell death induced by tamoxifen in breast cancer cell lines. *Evidence-Based Complementary and Alternative Medicine*.

[15] Jaganathan S. K., Mandal M. (2010). Involvement of non-protein thiols, mitochondrial dysfunction, reactive oxygen species and p53 in honey-induced apoptosis. *Investigational New Drugs*.

[16] Wen C. T. P., Hussein S. Z., Abdullah S., Karim N. A., Makpol S., Yusof Y. A. M. (2012). Gelam and nenas honeys inhibit proliferation of HT 29 colon cancer cells by inducing DNA damage and apoptosis while suppressing inflammation. *Asian Pacific Journal of Cancer Prevention*.

[17] Aljadi A. M., Yusoff K. M. (2003). Isolation and identification of phenolic acids in Malaysian honey with antibacterial properties. *Turkish Journal of Medical Sciences*.

[18] Kadan S., Saad B., Sasson Y., Zaid H. (2013). In vitro evaluations of cytotoxicity of eight antidiabetic medicinal plants and their effect on GLUT4 translocation. *Evidence-Based Complementary and Alternative Medicine*.

[19] Pyrzynska K., Biesaga M. (2009). Analysis of phenolic acids and flavonoids in honey. *TrAC Trends in Analytical Chemistry*.

[20] Manach C., Scalbert A., Morand C., Rémésy C., Jime L. (2018). Polyphenols: food sources and bioavailability. *The American Journal of Clinical Nutrition*.

[21] Afroz R., Em T., Zheng W., Pj L. (2016). Molecular pharmacology of honey. *Journal of Clinical and Experimental Pharmacology*.

[22] Cabras P., Angioni A., Tuberoso C. (1999). Homogentisic acid: a phenolic acid as a marker of strawberry-tree (*Arbutus unedo*) honey. *Journal of Agricultural and Food Chemistry*.

[23] Dong R., Zheng Y., Xu B. (2013). Phenolic profiles and antioxidant capacities of chinese unifloral honeys from different botanical and geographical sources. *Food and Bioprocess Technology*.

[24] Beretta G., Granata P., Ferrero M., Orioli M., Facino R. M. (2005). Standardization of antioxidant properties of honey by a combination of spectrophotometric/fluorimetric assays and chemometrics. *Analytica Chimica Acta*.

[25] Paramás A. M. G., Bárez J. A. G., Marcos C. C., García-Villanova R. J., Sánchez J. S. (2006). HPLC-fluorimetric method for analysis of amino acids in products of the hive (honey and bee-pollen). *Food Chemistry*.

[26] Andrade P., Ferreres F., Gil M. I., Tomás-Barberán F. A. (1997). Determination of phenolic compounds in honeys with different floral origin by capillary zone electrophoresis. *Food Chemistry*.

[27] Ferreres F., García-Viguera C., Tomás-Lorente F., Tomás-Barberán F. A. (1993). Hesperetin: A marker of the floral origin of citrus honey. *Journal of the Science of Food and Agriculture*.

[28] Tomás-Barberán F. A., Martos I., Ferreres F., Radovic B. S., Anklam E. (2001). HPLC flavonoid profiles as markers for the botanical origin of European unifloral honeys. *Journal of the Science of Food and Agriculture*.

[29] Yim S.-H., Kim H. J., Park S.-H. (2012). Cytotoxic caffeic acid derivatives from the rhizomes of Cimicifuga heracleifolia. *Archives of Pharmacal Research*.

[30] Rajendra Prasad N., Karthikeyan A., Karthikeyan S., Venkata Reddy B. (2011). Inhibitory effect of caffeic acid on cancer cell proliferation by oxidative mechanism in human HT-1080 fibrosarcoma cell line. *Molecular and Cellular Biochemistry*.

[31] Haslam E. (1996). Natural polyphenols (vegetable tannins) as drugs: possible modes of action. *Journal of Natural Products*.

[32] Khan N. S., Ahmad A., Hadi S. M. (2000). Anti-oxidant, pro-oxidant properties of tannic acid and its binding to DNA. *Chemico-Biological Interactions*.

[33] Yoon C.-H., Chung S.-J., Lee S.-W., Park Y.-B., Lee S.-K., Park M.-C. (2013). Gallic acid, a natural polyphenolic acid, induces apoptosis and inhibits proinflammatory gene expressions in rheumatoid arthritis fibroblast-like synoviocytes. *Joint Bone Spine*.

[34] Pérez R. A., Sánchez-Brunete C., Calvo R. M., Tadeo J. L. (2002). Analysis of volatiles from Spanish honeys by solid-phase microextraction and gas chromatography-mass spectrometry. *Journal of Agricultural and Food Chemistry*.

[35] Abdulkhaleq L. A., Assi M. A., Noor M. H. M., Abdullah R., Saad M. Z., Taufiq-Yap Y. H. (2017). Therapeutic uses of epicatechin in diabetes and cancer. *Veterinary World*.

[36] Islam M. R., Pervin T., Hossain H., Saha B., Hossain S. J. (2017). Physicochemical and antioxidant properties of honeys from the sundarbans mangrove forest of bangladesh. *Preventive Nutrition and Food Science*.

[37] Perna A., Intaglietta I., Simonetti A., Gambacorta E. (2013). A comparative study on phenolic profile, vitamin C content and antioxidant activity of Italian honeys of different botanical origin. *International Journal of Food Science & Technology*.

[38] Andrade P., Ferreres F., Teresa Amaral M. (1997). Analysis of honey phenolic acids by HPLC, its application to honey botanical characterization. *Journal of Liquid Chromatography & Related Technologies*.

[39] Biesaga M., Pyrzynska K. (2009). Liquid chromatography/tandem mass spectrometry studies of the phenolic compounds in honey. *Journal of Chromatography A*.

[40] Khalil M. I., Alam N., Moniruzzaman M., Sulaiman S. A., Gan S. H. (2011). Phenolic acid composition and antioxidant properties of malaysian honeys. *Journal of Food Science*.

[41] Kassim M., Achoui M., Mustafa M. R., Mohd M. A., Yusoff K. M. (2010). Ellagic acid, phenolic acids, and flavonoids in Malaysian honey extracts demonstrate in vitro anti-inflammatory activity. *Nutrition Research*.

[42] De Almeida A. M. M., Oliveira M. B. S., Da Costa J. G., Valentim I. B., Goulart M. O. F. (2016). Antioxidant capacity, physicochemical and floral characterization of honeys from the northeast of Brazil. *Revista Virtual de Química*.

[43] Afroz R., Tanvir E., Paul S., Bhoumik N. C., Gan S. H., Khalil M. I. (2015). DNA damage inhibition properties of sundarban honey and its phenolic composition. *Journal of Food Biochemistry*.

[44] Jaganathan S. K., Mandal M. (2009). Honey constituents and their apoptotic effect in colon cancer cells. *Journal of ApiProduct ApiMedical Science*.

[45] Henriques A., Jackson S., Cooper R., Burton N. (2006). Free radical production and quenching in honeys with wound healing potential. *Journal of Antimicrobial Chemotherapy*.

[46] Patisaul H. B., Jefferson W. (2010). The pros and cons of phytoestrogens. *Frontiers in Neuroendocrinology*.

[47] Tsiapara A. V., Jaakkola M., Chinou I. (2009). Bioactivity of Greek honey extracts on breast cancer (MCF-7), prostate cancer (PC-3) and endometrial cancer (Ishikawa) cells: profile analysis of extracts. *Food Chemistry*.

[48] Kyselova Z. (2011). Toxicological aspects of the use of phenolic compounds in disease prevention. *Interdisciplinary Toxicology*.

[49] Amiot M. J., Aubert S., Gonnet M., Tacchini M. (1989). Les composés phénoliques des miels : étude préliminaire sur l'identification et la quantification par familles. *Apidologie*.

[50] Rao P. V., Krishnan K. T., Salleh N., Gan S. H. (2016). Biological and therapeutic effects of honey produced by honey bees and stingless bees: A comparative review. *Revista Brasileira de Farmacognosia*.

[51] Ruiz-Ruiz J. C., Matus-Basto A. J., Acereto-Escoffié P., Segura-Campos M. R. (2017). Antioxidant and anti-inflammatory activities of phenolic compounds isolated from Melipona beecheii honey. *Food and Agricultural Immunology*.

[52] Ziegler R. G. (2004). Phytoestrogens and breast cancer. *American Journal of Clinical Nutrition*.

